# Exploring effort–reward imbalance and professional quality of life among health workers in Cape Town, South Africa: a mixed-methods study

**DOI:** 10.1186/s41256-022-00242-6

**Published:** 2022-03-01

**Authors:** N. Jensen, C. Lund, Z. Abrahams

**Affiliations:** 1 Department of Global Health and Social Medicine, King’s College London, London, UK; 2 Health Service and Population Research Department, Centre for Global Mental Health, Institute of Psychiatry, Psychology and Neuroscience, King’s Global Health Institute, King’s College London, London, UK; 3 Department of Psychiatry and Mental Health, Alan J Flisher Centre for Public Mental Health, University of Cape Town, Cape Town, South Africa

**Keywords:** South Africa, Health workers, Health system strengthening, Task shifting, Task sharing, Community health workers, PHC, Effort–reward imbalance, PROQOL, Equity, Common mental disorders

## Abstract

**Background:**

In the context of a growing appreciation for the wellbeing of the health workforce as the foundation of high-quality, sustainable health systems, this paper presents findings from two complementary studies to explore occupational stress and professional quality of life among health workers that were conducted in preparation for a task-shifting intervention to improve antenatal mental health services in Cape Town.

**Methods:**

This mixed-methods, cross-sectional study was conducted in public sector Midwife Obstetric Units and associated Non-Profit Organisations in Cape Town. Semi-structured interviews and a quantitative survey were conducted among facility-and community-based professional and lay health workers. The survey included demographic as well as effort–reward imbalance (ERI) and professional quality of life (PROQOL) questionnaires to examine overall levels of work-related psychosocial stress and professional quality of life, as well as differences between lay and professional health workers. Qualitative data was analysed using a thematic content analysis approach. Quantitative data was analysed using STATA 12.

**Results:**

Findings from 37 qualitative interviews highlighted the difficult working conditions and often limited reward and support structures experienced by health workers. Corroborating these findings, our quantitative survey of 165 professional and lay health workers revealed that most health workers experienced a mismatch between efforts spent and rewards gained at work (61.1% of professional and 70.2% of lay health workers; *p* = 0.302). There were few statistically significant differences in ERI and PROQOL scores between professional and lay health workers. Although Compassion Satisfaction was high for all health worker groups, lay health workers also showed elevated levels of burnout and compassion fatigue, with community-based health workers particularly affected.

**Conclusions:**

Findings of this study add to the existing evidence base on adverse working conditions faced by South African public-sector health workers that should be taken into consideration as national and local governments seek to ‘re-engineer’ South Africa’s Primary Health Care system. Furthermore, they also highlight the importance of taking into consideration the wellbeing of health workers themselves to develop interventions that can sustainably foster resilient and high-quality health systems.

**Supplementary Information:**

The online version contains supplementary material available at 10.1186/s41256-022-00242-6.

## Background

As a central ‘building block’ of health systems, health workers are a primary target of health system strengthening (HSS) interventions. Task shifting and task sharing—the redistribution of health service tasks among health workers, typically involving the shifting to/sharing of tasks with less specialised health worker cadres or lay health workers—have become major pillars in efforts to strengthen under-resourced and -performing health systems; often seen as a key strategy to engender better and more equitable access to health services especially in low-and middle-income countries (LMIC) [1–4]. According to several Systematic Reviews, task shifting interventions may indeed improve access to and outcomes from selected health services [5–7]; and yet, important questions remain, including about the impact of task shifting initiatives on health workers themselves [8].

Recent years have seen a growing appreciation for the wellbeing of the health workforce as the foundation of high-quality, sustainable health systems [9–11]. This is not least due to a growing body of evidence on the link between health workers’ perceptions of their working conditions and job performance, staff turnover, quality of care, patient outcomes, as well as their own health [12–16]. As a result, researchers have become increasingly interested in examining how global and national health priorities, policies and research programmes impact on those tasked with carrying them out [17, 18].

In the global health field, interest in Community Health Workers (CHWs) to deliver programmes has seen a significant resurgence in recent years, fuelled by hopes that they can mitigate enduring health worker shortages, improve access to care, and provide a crucial link between formal and informal care systems [19, 20]. In South Africa, CHWs are considered an increasingly central pillar of the country’s healthcare system and key to the government’s goal of reaching universal health coverage. This has been accompanied by efforts to consolidate existing community-based lay health worker programs, which have a long history in South Africa but proliferated during the country’s struggle against the HIV (Human Immunodeficiency Virus)/Aids epidemic [21–23].

In 2011, the South African government initiated its ‘Re-engineering of Primary Health Care’ scheme, with the aim to streamline and expand CHW programmes and integrate them more firmly within the public health system [24]. This includes consolidating CHW roles and employing them directly, rather than through government-funded non-profit organisation (NPO) subcontractors, as well as turning CHWs into more generalised health workers with responsibilities for a growing range of health promotion, prevention and treatment interventions [23, 25]. However, this has also raised concerns not just about the practicability of reorienting existing community-based care structures, but also about a growing disjuncture between the government’s emphasis on the importance of community-based care—and the often-precarious working conditions faced by CHWs, who are typically employed on low-paid, short-term contracts with limited support and opportunities for career progression [23, 26, 27].

Using instruments such as the stress factor scales and the Maslach Burnout Inventory or Professional Quality of Life (PROQOL) subscale, a number of existing studies have highlighted the high prevalence of work-related stress and burnout within in the South African health workforce [28–31]. Similarly, studies have sought to measure effort–reward-imbalance (ERI) among health workers. First proposed by German medical sociologist Johannes Siegrist [32], the ERI model posits that a failed reciprocity between efforts spent and rewards gained at work results in experiences of psychosocial stress and may lead to adverse health outcomes. The ERI model has been widely used to in occupational settings to identify psychosocial stress among workers, including health workers [32, 33]. However, despite concerns over the steady expansion of job roles and inadequate remuneration and reward structures, far fewer such studies have investigated work-related stress amongst lay health workers [34, 35]. To the best of our knowledge, no studies have used the ERI scale to examine work-related stress amongst health workers in South Africa.

This paper reports findings from two studies that were conducted before the implementation of a task-shifting intervention involving lay health workers to improve the detection and treatment of Common Mental Disorders (CMDs) and/or experiences of domestic violence among pregnant mothers from underserved communities in Cape Town. Efforts to improve the public-sector provision of mental health support for pregnant mothers respond to a growing burden of mental health disorders in South Africa [36], inequities in prevalence and service provision [37], and recent national efforts to better integrate mental health services into PHC [38]. They also take place in a context of wider efforts by the South African government to formalise and significantly expand the roles of community-based lay health workers.

In light of the shifting dynamics of South Africa’s healthcare system and to guide the design of our planned intervention, the first phase of our research project involved mapping the existing organisation of maternal health services in the study area, including through 37 semi-structured interviews with maternal health service providers. These interviews revealed a number of potential barriers to the implementation of a successful screening and treatment intervention, including concerns over already high workloads [33].

In response to these findings, a second study was conducted to investigate the work-related psychosocial wellbeing and quality of life of professional and lay health workers before the implementation of a task shifting intervention which would extend their job descriptions to include a mental health component. We also hypothesized that lay health workers may experience higher effort–reward imbalance and lower quality of life compared to professional health workers.

## Methods

### Setting and study design

In South Africa, free antenatal care is provided at Midwife Obstetric Units (MOUs) or basic antenatal care (BANC) clinics by professional nurses. These facilities frequently include onsite specialised staff, such as psychiatric nurses and/or social workers, as well as lay health workers, including HIV and breastfeeding counsellors. In addition, patients who attend facilities receive follow-up community-based support from lay health workers, who are largely employed by Department of Health (DoH)-funded NPO subcontractors and managed by a cadre of professional nurses, called outreach team leaders (OTL).

As part of the Health System Strengthening in sub-Saharan Africa (ASSET) study [39], researchers worked with managers from the Cape Metropolitan health district within the Western Cape DoH to develop an intervention for the detection, referral and treatment of common mental disorders (CMDs) and/or experiences of domestic violence among pregnant mothers attending MOU and BANC facilities. The intervention involves the screening by facility-based nursing staff of all pregnant women for CMD and experiences of domestic violence during their antenatal visits. Those who screen positive are offered three sessions of problem-solving therapy delivered by community health workers (CHWs) in the pregnant women’s homes [40].

This article reports on findings from research that was conducted at baseline, that is before the implementation of a planned cluster randomised control trial at MOUs and BANC clinics situated in the ‘Cape Flats’—an area made up of low-income communities on the outskirts of the city of Cape Town that emerged as a result of forced relocations to informal settlements during Apartheid [41].

In a first step, interviews were conducted with 37 health workers at four MoUs and associated NPOs. The interviews were aimed at establishing existing perinatal mental health workflows and referral pathways. Furthermore, health workers were asked about their role in—and attitude to—detecting, referring and treating pregnant women with symptoms of depression or anxiety, and experiences of domestic violence, as well as about the acceptability and feasibility of a routine screening and counselling service for pregnant women. Findings on the perceived facilitators and barriers to the detection and treatment of pregnant women with symptoms of CMDs and experiences of domestic violence were reported elsewhere [42]. For this article, we draw on expanded interview material to highlight some of the concerns specifically raised in relation to work environments.

In response to concerns raised by initial findings from the qualitative interviews, it was decided to add an additional study component to assess effort reward imbalance and professional quality of life of health workers at baseline. A quantitative survey was conducted among both professional and lay health workers, which included demographic as well as effort–reward imbalance (ERI) and professional quality of life (PROQOL) questionnaires.

### Data collection and tools

#### Qualitative interviews

Between November 2018 and July 2019, in-depth semi-structured interviews were conducted with 25 facility-based health workers providing care to perinatal women at four MOUs, as well as 12 health workers working for NPOs that support the MOUs through the provision of community-based care. The following cadres of health workers were identified and invited to participate in interviews: operational managers, antenatal care (ANC) nurses, breastfeeding counsellors, HIV counsellors, health promotion officers, mental health nurses, social workers, CHWs and OTLs. Facilities had one health worker from each cadre (except for ANC nurses, CHWs and OTLs) who were identified by their managers and invited to an interview. ANC nurses, OTLs and CHWs were identified by their managers for participation in the study, based on their availability on the day the interviews were conducted, and their seniority in the organisation. Health workers were told that participation in the study was entirely voluntary, and all but one health promotion officer agreed to be interviewed.

English language, semi-structured interview guides were developed and translated to Afrikaans and IsiXhosa by bilingual experts. The interviews were administered by trained researchers in English, Afrikaans and IsiXhosa, took between 30 and 60 min to complete, and were audio-recorded.

#### Quantitative survey

Between January and March 2020, subdistrict-, facility- and community-based health workers linked to seven randomly selected intervention facilities attended various cascaded training sessions in preparation for the intervention. Overall, 14 facilities were selected to participate in the cluster randomised controlled trial, including a combination of MOUs and BANC clinics that cover the four sub-districts of the Cape Town Metropolitan area. The 14 facilities were randomly allocated into either intervention or control arm. The sample size was based on the RCT design, powered to show a significant improvement in depression/anxiety outcomes among women attending antenatal care facilities in seven intervention and seven control facilities*.* Before commencement of each training session at the seven intervention facilities, an overview of the ASSET study was presented, and all participating health workers were asked to complete an anonymous, self-administered questionnaire. Those who agreed to participate included community-based services (CBS) trainers, operational managers, antenatal care nurses, social workers, mental health nurses, health promotion officers, breastfeeding counsellors, HIV counsellors, CHWs, OTLs and NPO coordinators. In addition to a questionnaire collecting basic demographic and occupational characteristics, participants were asked to complete the short (16-item) version of the Effort-Reward Imbalance (ERI) Scale, as well as the Professional Quality of Life (PROQOL) scale.

The Effort-Reward Imbalance (ERI) model comprises three psychometric scales: effort, reward, and overcommitment. The psychometric properties of the ERI scale have been extensively tested and its reliability and validity have shown to be satisfactory, including for the short version of the scale [43, 44]. Whereas most ERI research has been conducted in higher-income countries, one of the few studies that employed the ERI scale among African health workers demonstrated a satisfactory consistency between the three internal scales [45].

For this study, we used the shorter (16-item) version of the ERI questionnaire [44], which we employed without alteration (Additional file 1). Health workers were asked to complete a self-administered English/Afrikaans or English/IsiXhosa questionnaire, with Afrikaans and IsiXhosa translations provided alongside the English wording. In this version, ‘effort’ is measured by three 4-point Likert-scale items (ERI 1–3) that pertain to the demands experienced in the workplace, such as relating to workload and level of responsibility (example item: ‘I have constant time pressure due to a heavy workload’). Scores of the effort sub-scale range from three to a maximum of 12, with higher scores indicating higher level of demands. ‘Reward’ is measured by seven Likert-scale items (ERI 4–10) that are further divided into three sub-scales for ‘Esteem’, ‘Promotion’ and ‘Security’ (example item: ‘I receive the respect I deserve from my superior or a respective relevant person’). Below we report both, the total reward score (ranging from 7 to 28) and the scores for the three sub-scales, with lower scores indicating lower perceived rewards. The ‘Overcommitment’ scale includes six Likert-scale items (ERI 11–16) with scores ranging from 6 to 24 (example item: ‘When I get home, I can easily relax and ‘switch off’ work’).

Higher mean scores for ‘Effort’ and ‘Overcommitment’ and lower mean scores for ‘Reward’ are an indicator of perceived psychosocial stress in the workplace [43]. Further indication of the perceived stress is the effort–reward ratio (ER ratio), which quantifies the imbalance between effort and reward. For all study participants, we computed the ER ratio by sum ‘Effort’ scores as numerator and sum ‘Reward’ scores as denominator multiplied by a correction factor of 7/3 to account for the unequal number of items for each scale (ER ratio = kE/R) [39]. An ER ratio < 1 indicates a low perceived effort–reward imbalance, an ER ratio > 1 indicates a high level of perceived effort–reward imbalance and a high level of psychosocial stress in the workplace [39].

The professional quality of life (ProQOL) scale is widely used to measure positive and negative elements of the professional quality of life of people in helping professions, such as health workers [46, 47]. Developed by Figley and Stamm, it uses three subscales: Compassion Satisfaction (CS) aims to measure the positive aspects of providing help (example item: ‘I get satisfaction from being able to help people’); negative outcomes are captured by Compassion Fatigue (CF)—sometimes also referred to as Secondary Traumatic Stress (STS) (example item: ‘I think that I might have been affected by the traumatic stress of those I helped’)—and Burnout (BO) (example item: ‘I feel trapped by my job’). The three subscales are scored separately and no cumulative score is derived [48]. For each subscale, a score above 42 is considered high, 23–41 average, and 22 or less is considered low [46]. The ProQOL scale has been widely used, including in multiple studies in healthcare settings in South Africa [49–52].

### Data analysis

#### Qualitative interviews

The semi-structured interviews that were conducted in English were transcribed verbatim, while interviews conducted in Afrikaans or IsiXhosa were translated into English and transcribed by bilingual speakers. Transcripts were analysed using a thematic analysis approach to generate initial codes and define, search for and review themes [53]. The development of initial codes was guided to a certain degree by the semi-structured interview topics. Further themes not captured by the initial coding were identified through extensive reading of the transcripts and coding passages interpreted as important. Transcripts and data were managed using NVivo 12 Pro qualitative data analysis software (QSR International Pty Ltd) [54].

This present analysis is based on the original content analysis but further elaborates on specific findings related to the job roles of interviewees as well as interviewees’ narratives of stressful work environments.

#### Quantitative survey

Quantitative data analysis was performed using STATA 12 for Mac. Participants with incomplete data were excluded from the analysis. For the ERI questionnaire, mean scores and standard deviations were calculated for the three individual scale scores (Effort, Reward, Overcommitment), as well as three Reward subscales (Esteem, Promotion, Security). Mean scores and standard deviations were also calculated for the PROQOL subscale scores for compassion satisfaction, burnout and secondary trauma/compassion fatigue. Categorical variables were described using frequency and percentages, and associations measured using Chi-square tests. Continuous variables were described using means and standard deviations, and associations measured using t-tests. A *p* value of < 0.05 was considered statistically significant.

For the purpose of our statistical analysis, surveyed health workers were divided into five categories: facility-based professional health workers, facility-based lay health workers, community-based professional health workers, community-based lay health workers, and sub-structure health workers. In doing so, we followed a widely used definition by Lewin et al. [55] of lay health workers as “any health worker carrying out functions related to health care delivery; trained in some way in the context of the intervention; and having no formal professional or paraprofessional certificated or degreed tertiary education” (p. 1). Table 1 provides an overview of the five categories we used and the types of health workers they comprise.

**Table 1:** Classification of health workers according to level of qualification and workplace

	Facility-based	Community-based	Sub-structure-based
Professional	Operational manager	NPO^a^ manager	CBS^d^ trainer
	Nurses	OTL^b^	
	Enrolled nurse		
	Mental health nurse		
	Social worker		
Lay	Nursing assistant	CHW^c^ supervisor	
	Health promoter	CHW	
	HIV counsellor		
	Breastfeeding counsellor		

^a^Non-profit organisation manager

^b^Outreach team leader

^c^Community health worker

^d^Community-based services

### Ethical approval

Ethical approval for the study was obtained from the Human Research Ethics Committee at the University of Cape Town (Ref No: 139/2018) and the Psychiatry, Nursing and Midwifery Research Ethics Subcommittee at King’s College London (Ref No: 17/18-7807). In addition, the Western Cape Department of Health approved the use of the research sites (Ref No: WC_201807_008). Those who participated in the study did so voluntarily and provided written, informed consent. All participants were informed that they were free to withdraw from the study at any time without consequences. No financial incentives were provided.

## Results

### Qualitative interviews

Key themes identified in the interviews pertained to health workers roles and responsibilities, their knowledge of and experience with mental health and violence, their attitude towards the feasibility of the proposed screening and counselling intervention, as well as their perceptions of positive and negative aspects of their work. As previously noted, headline interview findings about interviewees’ perceived facilitators and barriers to the implementation of a task shifting intervention were reported elsewhere [40]. Below, we specifically present findings that pertain to the job roles of professional and lay health workers involved in the care of pregnant people, as well as the challenging work environments that these health workers operate in.

#### Demographic and occupational characteristics

Semi-structured interviews were conducted with 37 service providers from four MOUs and associated NPOs. As Table 2 shows, interviewees were predominantly female (91.9%) and older (72.9% aged 40 or older), and the majority self-identified as ‘coloured’ (75.5%). Interviewees included both staff directly involved in care, such as nurses, and staff in managerial positions, such as managers of NPOs. The majority of interviewees worked at a facility (67.5%) and were classified as professional health workers (62.1%).

**Table 2:** Demographic and occupational characteristics of interviewees

Characteristics	Health workers (n = 37) n (%)
*Age*	
< 30	5 (13.5)
30–40	5 (13.5)
41–50	14 (37.8)
> 50	13 (35.1)
*Gender*	
Female	34 (91.9)
Male	3 (8.1)
*Ethnicity*	
Coloured	28 (75.7)
Black South African	6 (16.2)
White	3 (8.1)
*Health worker role*	
Manager	13 (35.1)
Professional health worker	10 (27.0)
Lay health worker	14 (37.9)
*Employment environment*	
Facility-based	25 (67.5)
Community-based	12 (32.5)

Since our study sought to examine potential differences in occupational psychosocial stress and professional quality of life between professional and lay health workers, we assigned all interviewees and survey participants according to these categories, with a further differentiation made based on if they worked in a PHC facility or in the community. The terminological distinction between professional and lay health workers pervades the global health literature and is typically proposed to rest on the restricted range of tasks performed by lay health workers as well as their lack of professional or tertiary education. In practice, however, we found this distinction between lay and professional much more difficult to uphold.

One the one hand, healthcare delivery in South Africa has long involved various ‘substitute health workers’ [56], formally recognised health worker cadres who perform tasks usually reserved for internationally recognised professionals such as doctors and nurses. For example, following a series of reforms to nursing education, there co-exist today a number of older and newer categories of nurses in South Africa, including professional nurses with a four-year diploma or baccalaureate degree, staff nurses with a 3-year diploma, enrolled nurses with 2-year certificate from nursing college, and enrolled nursing auxiliaries or nursing assistants with 1-year training certificate [57]. On the other hand, the progressive formalisation of lay health workers in South Africa has both expanded their roles and brought further training and promotion opportunities. For example, ‘CHW supervisors’ tend to have no tertiary education—and are hence considered lay health workers—and yet, they have typically obtained additional training and certificates and have similar supervisory responsibilities to Outreach Team Leaders (OTLs), who are considered professional health workers.

For our classification, we relied on a combination of level of education and job title to categorise both CHW supervisors and facility-based lay counsellors as lay health workers, even though some had formally recognised certifications. But our findings also suggest that the pervasive dichotomisation between lay and professional health workers based on job roles and levels of education is somewhat tenuous. That this raises a series of important questions regarding existing reward structures, especially in contexts like South Africa where reform programs continue to expand lay health workers’ roles and responsibilities, will be further discussed below.

#### Job roles

Our qualitative interviews helped to further clarify the organisation of antenatal care in the Western Cape province and the role that different types of service providers play, or may play, in the provision of care.

Public-sector antenatal care is an integral part of PHC and provided through BANC (Basic Antenatal Care) clinics or Midwife Obstetric Units (MOUs) in Community Health Centres. MOUs are run by midwives, who are professional nurses with additional midwifery qualifications, and antenatal services are typically provided by professional nurses, enrolled nurses, nursing assistants and other lay health workers, and a visiting or residential medical officer. At least some clinics and health centres have further specialised support staff, such as dieticians and social workers, that may get involved in aspects of maternity care. Despite the plans to systematise and centralise PHC as part of South Africa’s ‘Re-engineering PHC’ scheme, the Department of Health still subcontracts a multitude of NPOs to provide additional care services, often both at facility and at community level via a referral system. Indeed, our interviews revealed a highly complex service delivery landscape, whereby facilities are often supported by network of NPOs, which, in turn, support a network of facilities and their surrounding areas.

NPOs are usually run by managers with professional nursing degrees, whose responsibilities include the day-to-day running of NPOs, ensuring adherence to and reporting of performance criteria set by the DoH, and ensuring continuous funding for their organisation. Although NPOs often have long-established relationships with certain facilities, their contracts with the DoH tend to be relatively short, sometimes only lasting 1 year. As some NPO managers suggested in our interviews, this added to their administrative burden, with considerable time spent on auditing procedures and writing new business plans to ensure renewal of funding. In their day-to-day running of community-based services, NPO managers are supported by Coordinators, typically either fully-trained or enrolled nurses, who are responsible for the training and supervision of lay health workers. Coordinators often oversee a sizeable number of both facility- and community-based lay health workers.

#### Challenging working environments

Although our interviews were not specifically designed to explore workplace challenges, such challenges nonetheless emerged in many of our interviews as health workers reflected on their current job roles and the feasibility of improving antenatal mental health services through a task shifting intervention.

##### Lack of standardised and regulated support structure for lay health workers

Our interviews suggested highly uneven levels of supervision and support provided to/received by lay health workers. Whereas some interviewees noted the existence of regular supervision, including supervision of CHWs’ home visits, monthly or bi-monthly supervision meetings seemed much more common, with meetings often focused on the discussion of ‘stats’—the daily and monthly client quotas that lay health workers are expected to meet and that NPOs are required to report to the DoH on a regular basis.

In addition to a seeming lack of standardised and regulated support structures, our interviews also highlighted a lack of consistency when it came to the roles fulfilled by lay health workers employed through NPOs: among those working largely in the community, some were referred to as ‘home-based carers’, with responsibilities that included general care responsibilities, such as wound dressing, chronic medication distribution and palliative care. There were also lay health workers who worked as adherence counsellors to offer a range of community support specifically to individuals with drug-resistant tuberculosis and/or HIV; and community health workers employed through specific maternal and child health programmes with responsibilities including health promotion and prevention, and also “integrated work like postnatal and all the other stuff” (NPO Coordinator 1).

In addition to community-based lay health workers, NPOs also used to be tasked with overseeing lay health workers that were, at the time of our interviews, more or less permanently based at specific health facilities (9% of respondents), such as HIV counsellors, ‘Integrated’ counsellors delivering both TB and HIV/AIDS counselling, and breastfeeding counsellors. However, as part of ‘Re-engineering PHC’, efforts have been ongoing to phase out such facility-based lay health worker roles and streamline community-based care through the creating of a generalised CHW role. One NPO manager noted the concerns that these pending changes were causing among some lay health workers: “I think there’s a bit of fear going around if we integrate (facility-based lay health workers with CHWs), are we gonna be too many and then does it mean that we might lose our jobs because there’s not enough work… So there’s a bit of resistance there. Uhm the Department [of Health] is aware of this resistance but it’s, yah, the pressure, it’s just completely taken off.” (NPO Coordinator).

Whereas the planned changes are aimed at standardising and better regulating the position of lay health workers, some interviewees also highlighted the challenges of managing this transition: “So the Department [of Health] is looking at having us integrate the two teams [of adherence counsellors and home-based carers] but they are completely… although they work the same hours, in term of salary it’s the same, but they do completely different work. Not all of our [adherence counsellors] are home-based trained, so we can’t utilise them in our home-based care programme. So we are currently having challenges with getting a service provider to provide accredited home based care training. So that is delaying this process from happening.” (NPO Coordinator 2).

##### Lay health workers’ responsibilities and remuneration

We previously reported on high workloads and patient numbers that emerged as a major concern among interviewed nurses as a potential barrier to our task shifting intervention [33]. But our interviews also highlighted that lay health workers are exposed to similarly pressured environments. For example, one facility-based professional health worker voiced their concern about asking CHWs to take on even more tasks, especially if this did not involve additional supervision and compensation: “[…] it is not going to be fair saying that we are doing… –putting a CHW in a position, you educate her, they get the training and everything with regards to counselling, what to look out for and that, and they are not… –already now, the CHWs earn little, you understand? […] So, we do not want them to be at the end of the day put in a situation and then they take it further without supervision because it is almost like they are the doctor, sister, counsellor, social worker, everything when they step in that house” (facility-based Social Worker). Indeed, interviews suggested that the average monthly salary for CHWs is around 4000–4500 South African Rand (US$275–310) per month for an 8-h workday, barely enough to cover basic living costs in the Western Cape Metropolitan region.

At the time of our interviews, facility-based lay health workers were predominantly employed on part-time contracts and typically worked only half-day shifts. Some interviewees identified this as a key obstacle to these health workers’ ability to take on additional tasks. As one CHW Coordinator noted: “I do not know if they will have time to actually assist those people because their time. They only work half days already […] So I think if the government can maybe pay more people…” (CHW Coordinator).

On the other hand, however, there were suggestions that fewer working hours also had a protective effect on the levels of stress that community-based health workers were exposed to: “So, in general you do not have a huge feeling of burn out amongst your staff where they feel they are tired, they cannot cope, they… I think the 4.5 h they work is enough. If they were working eight hours and the distance they supposed to walk from and here again to sign out of these things then they will be tired” (CHW Coordinator). And yet, during the course of our study, the DoH also introduced a first set of changes to the working conditions of lay health workers, which included the extension of working hours to 8-h shifts.

##### Tension between material and non-material job rewards

Despite the perceived challenges, many interviewees emphasised their ‘passion’ or ‘love’ for their jobs. One facility-based lay health worker noted that “[…] because I have a passion for people, I feel like I am a fish in the water, swimming and enjoying what I’m doing” (Health Promoter). Similarly, one CHW highlighted their connection to the community they worked in to note that: “The reason why I am working here is to make a difference in some people’s life” (Community Health worker 1). And yet, there was also a clear tension between health workers passion for helping people and the pressures they faced at work. As one CHW noted: “I love everything but it is tiring sometimes and it drains you” (Community Health worker 2). Another CHW comment highlights that lay health workers often stay in their jobs despite being unhappy with their salaries because of a deep commitment to their patients: “At night I go and lay down and then think about the whole day but when you are tired like that, you say, ‘yoh, enough is enough, I will not work anymore there because the money is not enough’. But then when I… after that when I think ‘no but what is going to happen to my patient?’, I know that one is waiting for me” (CHW 3).

##### Exposure to violence

Lastly, a notable additional challenge that emerged in our interviews is the high level of violence in communities. Several interviewees related incidents of violence, including knife attacks and shootings, that they or their colleagues had been exposed to. One CHW Coordinator expressed concern about the ‘desensitising’ effects of working under such dangerous conditions, emphasising that CHWs “work under terrible conditions […] I wouldn’t want to work under those conditions ever” (CHW Coordinator). In response to safety concerns, several NPOs that we spoke to reported preferring CHWs to conduct home visits in pairs, although one Coordinator also pointed out that, if CHWs visited clients together, this also made it more difficult to meet the targets set by the DoH: “it’s one person’s stats, so it even creates more pressure doubling up” (CHW Coordinator).

The rich data that emerged in our interviews about the often competing challenges and gratifications that health workers experienced in their work environment prompted us to conduct a quantitative survey to systematically assess the perceptions of psychosocial stress and professional quality of life among those health workers who would be tasked with implementing the planned task shifting intervention.

### Quantitative survey

#### Demographic and occupational characteristics

Table 3 presents the demographic characteristics of the 165 health workers who completed the survey. The vast majority of health workers were 30 years old or older (91.8%), female (95.1%), identified as Black South African (67.3%) and listed IsiXhosa as their first language (63.6%).

**Table 3:** Demographic characteristics of survey participants

Characteristics	Health workers (n = 165) n (%)
*Age*	
< 30	12 (8.2)
30–40	49 (33.3)
41–50	46 (31.3)
> 50	40 (27.2)
*Gender*	
Female	156 (95.1)
Male	8 (4.9)
*Home language*	
English	29 (17.6)
Afrikaans	28 (17.0)
IsiXhosa	105 (63.6)
Other African language	3 (1.8)
*Ethnicity*	
Coloured*	50 (30.3)
Black South African	111 (67.3)
Black African	4 (2.4)
*Household size*	
1–2	28 (17.0)
3–5	102 (61.8)
> 5	35 (21.2)
*Has a partner*	115 (70.6)
*Number of children*	
0–1	43 (26.1)
2–4	98 (59.4)
> 4	24 (14.6)
*Mode of transport to work*	
Public transport	41 (24.9)
Lift club	3 (1.8)
Own car	39 (23.6)
Walk	82 (49.7)

^*^Individuals of mixed ethnic ancestry [58]

Table 4 presents the occupational characteristics of survey respondents. The majority of survey respondents were lay health workers (73.4%) that were either facility- (10.7%) or community-based (89.3%), with Grade 11–12 as the highest level of education (high school graduation). Professional health workers (26.7% of respondents) included managers of NPOs and facility-based professional health workers, who were all educated to Diploma- or Degree-level. Survey respondents also included eight CBS trainers who delivered training sessions to CHWs on the ASSET counselling intervention and are employed by the ‘sub-district’, i.e. the provincial government.

**Table 4:** Occupational characteristics of health workers

Characteristics	Health workers (n = 165)		*P* value
	Professional (n = 44) n (%)	Lay (n = 121) n (%)	
*Employer*			
DoH^a^	21 (72.4)	8 (27.6)	< 0.001
NPO^b^	23 (16.9)	113 (83.1)	
*Work area*			
Facility-based	13 (29.6)	13 (10.7)	< 0.001
Community-based	23 (52.3)	108 (89.3)	
Sub-district	8 (18.2)	0	
*Education*			
< Grade 11	0	9 (7.5)	< 0.001
Grade 11–12	0	100 (83.3)	
Certificate	0	11 (9.2)	
Diploma	27 (64.3)	0	
Degree	11 (26.2)	0	
Honours	4 (9.5)	0	
*Time in position*			
< 2 years	13 (29.6)	18 (15.0)	0.012
2–5 years	16 (36.4)	52 (43.3)	
5–10 years	4 (9.1)	33 (27.5)	
> 10 years	11 (25.0)	17 (14.2)	

^a^Department of Health

^b^Non-profit organisation

#### Effort–reward imbalance

Table 5 displays the means and standard deviations of the ERI and PROQOL scales according to work area (facility vs. community-based) and level of professionalisation (professional vs. lay health workers) within those work areas.

**Table 5:** Psychosocial characteristics by health worker type

Characteristics	Facility-based health workers		*P* value*	Community-based health workers		*P* value*	Total		*P* value*
	Professional (n = 13) Mean (SD)	Lay (n = 13) Mean (SD)		Professional (n = 23) Mean (SD)	Lay (n = 108) Mean (SD)		Professional (n = 36) Mean (SD)	Lay (n = 121) Mean (SD)	
*Effort-reward imbalance (ERI) scores*									
Effort	8.38 (1.94)	9.33 (1.61)	0.198	8.35 (1.81)	8.17 (1.91)	0.699	8.36 (1.83)	8.29 (1.91)	0.854
Reward	18.62 (2.28)	17.23 (3.11)	0.280	18.48 (3.60)	17.41 (2.84)	0.124	18.53 (3.44)	17.40 (2.86)	**0.048**
Esteem	6.62 (1.33)	5.92 (1.38)	0.205	6.26 (1.57)	5.93 (1.36)	0.310	6.39 (1.48)	5.93 (1.36)	0.085
Promotion	7.50 (1.31)	7.36 (1.91)	0.843	8.00 (1.45)	7.70 (1.61)	0.436	7.52 (1.58)	7.67(1.63)	0.632
Security	5.27 (1.19)	4.38 (1.50)	0.128	4.68 (1.59)	4.63 (1.42)	0.875	4.87 (1.47)	4.60 (1.43)	0.329
Overcommitment	14.38 (1.89)	14.08 (2.56)	0.731	14.87 (2.05)	13.94 (2.28)	0.072	14.69 (1.98)	13.95 (2.30)	0.081
*Professional quality of life (PROQOL) scores*									
Compassion satisfaction	44.91 (3.18)	39.07(2.45)	**0.050**	42.56 (7.56)	41.34 (6.55)	0.488	43.45 (6.29)	41.03(6.90)	0.094
Burnout	21.67 (5.89)	22.09 (5.61)	0.871	20.05 (4.47)	23.26 (4.41)	**0.005**	20.57 (4.92)	23.12 (4.55)	**0.011**
Compassion fatigue	21.42 (5.16)	23.36 (4.67)	0.355	22.95 (4.89)	26.51(6.42)	**0.021**	22.38 (4.97)	26.17 (6.31)	**0.002**

Characteristics	Facility-based health workers		*P* value*	Community-based health workers		*P* value*	Total		*P* value*
	n (%)	n (%)		n (%)	n (%)		n (%)	n (%)	
*Effort-reward (ER) ratio based on ERI scores*									
Low effort–reward imbalance^a^	6 (46.2)	1 (7.7)	0.027**	8 (34.8)	35 (32.4)	0.826**	14 (38.9)	36 (29.8)	0.302**
High effort–reward imbalance^b^	7 (53.8)	12 (92.3)		15 (65.2)	73 (67.6)		22 (61.1)	85 (70.2)	

Values in bold indicate statistically significant results

^*^T-test for continuous variables

^**^Chi-squared test for categorical variables

^a^Low effort–reward imbalance—ratio of effort versus reward < 1

^b^High effort–reward imbalance—ratio of effort versus reward score > 1

There were only a few items for which we observed statistically significant differences in ERI scores between professional and lay health workers: overall results suggest that professional health workers gain more rewards from their work compared to lay health workers (18.5 vs. 17.4; although results were only borderline statistically significant; *p* = 0.048). This fits with a higher percentage of lay HCWs with high ER imbalance (> 1) than that of professional HCWs overall (70.2 vs. 61.1%). Although this difference between lay and professional health workers was not statistically significant (*p* = 0.302), our results indeed highlight the high levels of effort–reward imbalance experienced across all cadres of surveyed health workers, indicating a perceived mismatch between the effort put into their work and the rewards received in return. This mismatch was highest amongst facility-based lay health workers, amongst whom 92.3% had an ERI score above 1, compared to 53.8% of professional facility-based health workers (*p* = 0.027).

#### Professional quality of life

The ProQOL scores for Compassion Satisfaction, Burnout and Compassion Fatigue for facility-and community-based health workers are also shown in Table 5. Mean scores showed that lay health workers showed moderate levels of Burnout and Compassion Fatigue, compared to low levels for professional health care workers, with differences achieving statistical significance (*p* < 0.05). Notably, burnout and compassion fatigue levels were highest among community-based lay health workers. Interestingly, mean scores for Compassion Satisfaction were high for all professional and lay health workers apart from facility-based health workers, for whom the average score was moderate.

## Discussion

This mixed-methods study was part of the Diagnostic Phase of the ASSET research project on devising, implementing and evaluating a task-shifting intervention for the routine detection and treatment of CMD and domestic violence among pregnant women in Cape Town. Findings from semi-structured interviews suggest that while health workers are highly committed to their care of patients, they also described pressurised working environments in which high demands, caused by oftentimes high workloads and working in communities afflicted by a multitude of economic social difficulties, converge with a fragile support system. Indeed, our subsequently conducted survey revealed that—already at baseline, before the implementation of the ASSET intervention—there was a significant percentage of all health workers who experienced a mismatch between the efforts spent and rewards gained in their workplace. Whereas lay health workers overall reported less occupational rewards compared to professional heath workers, there was no statistically significant difference between lay and professional health workers in terms of Effort-Reward Imbalance. However, the embeddedness of CHWs within communities and exposure to the prevalent high levels of health and social problems, combined with an often-limited support system, may put them at greater risk of feeling overwhelmed, as is reflected in higher levels of burnout and compassion fatigue in this group found in our study.

The theoretical model behind the ERI scale posits that a mismatch between efforts spent and rewards gained in occupational environments elicits negative emotions and stress [33]. Efforts are conceived as the demands and obligations placed on workers, whereas rewards represent the economic and non-economic returns that workers expect, including financial rewards, career development opportunities and/or recognition and esteem [49]. Equity considerations are thus at the heart of the ERI model: put simply, an effort–reward imbalance exists where workers perceive to give more to their work than they receive back in return—a situation that Siegrist describes as ‘high cost/low gain’ [49]. As a third dimension, overcommitment refers to personal coping characteristics that can have a modifying influence: psychosocial stress may be particularly strong in those workers who also score high on overcommitment [49].

The ERI model can be used to highlight differences in job-related stress among health workers, according to demographic or occupational characteristics, such as age, gender and job role [59]. Our initial hypothesis that lay health workers may experience higher levels of effort–reward imbalance compared to professional staff was, in part, based on existing literature that has highlighted the difficult working conditions for lay health workers [23, 26, 60]. As part of the South African government’s ‘PHC Re-engineering’ scheme, lay health worker programmes are currently also undergoing significant changes that, even though they are aimed at strengthening PHC services and improving programme stability in the long-run, are accompanied by significant changes for lay health workers themselves.

With government plans to phase out the role of facility-based lay health workers, it is perhaps unsurprising that almost all survey respondents from this particular cadre reported high levels of effort–reward imbalance. The other key pillar of the government’s ‘Re-engineering PHC’ scheme envisions the creating of a larger cadre of more generalised CHWs. Other studies suggest that, in recent years, CHW roles have already significantly expanded to encompass an increasingly comprehensive range of health promotion, prevention and treatment tasks [27, 61]. The planned reorientation away from predominantly curative and disease-specific roles might help to streamline and systematise the training of and support provided to CHWs. At the same time, it may bolster the role of lay health workers as a ‘first line of support’ for especially underserved communities [62]. But our findings also add to concerns that there is a risk that CHWs become overburdened, especially if efforts to improve their working conditions do not keep pace with the expansion of their responsibilities. The South African Department of Health [63] has acknowledged that the ambiguous role of lay health workers means that “the system does not accord them the status of an employee, with the rights and benefits that come with it under South African labour law” (p. 14). If lay health workers are meant to play a central role in bolstering the PHC system, these issues will have to be urgently addressed.

And yet, our findings also suggests that this is not a problem that is limited to lay health workers. Indeed, contrary to our initial hypothesis that lay health workers may experience higher levels of effort reward imbalance, our study findings suggest that a majority of all PHC service providers included in our study experienced a mismatch in efforts spent and rewards gained. Whereas professional health workers seemed to gain more reward from their work—which may, in part, be linked to the higher esteem usually attached to professional positions (although slight differences in Esteem scores were not statistically significant in our survey)—they also reported high Effort scores.

Government efforts to ‘re-engineer’ PHC will therefore have to be managed carefully to avoid further exacerbating work-related stress and its negative consequences. Studies have long highlighted high levels of work dissatisfaction and burnout among South African health workers in South Africa [63–65]. There are some indications that dissatisfaction among public-sector health workers in South Africa caused by poor remuneration, high workloads, weak support structures and limited career development opportunities is at least to some extent mitigated by the satisfaction that health workers gain from their care work and being engaged in their communities [66]—findings that seem to be corroborated in our study by the many interviewees who spoke of their love and passion for their jobs. But our survey also revealed that high levels of Compassion Satisfaction among all health workers were accompanied by moderate levels of Burnout and Fatigue. This should act as a warning sign that gaining gratification from care work alone does not make for happy health workers and cannot replace the need for a healthy psychosocial work environment. Whereas expanding CHW programs may improve healthcare access while alleviating some of the pressure on the strained public health system, there is also a risk that this merely re-locates the problem towards an even more vulnerable cadre of workers.

Although this was not explicitly examined in our current study, there is an ever-growing body of evidence on the negative mental and physical health impacts caused by stressful work environments [14, 67, 68], and their role in explaining health inequalities [69]. Other studies conducted in healthcare settings have also pointed to a positive association between effort–reward-imbalance among health workers and their intention to leave their jobs [70] and poorer quality of care [71]. Since a key rationale for expanding CHW programs and task-shifting initiatives in South Africa is to mitigate the significant shortage of health workers and to improve health equity, our study highlights the need to ensure that workload allocations, supervision and remuneration for all health workers are carefully managed to avoid further exacerbating rather than mitigating the health worker crisis in South Africa.

In response to these findings, the ASSET Cape Town team further fine-tuned the design of the task-shifting intervention to ensure adequate supervision for CHWs tasked with delivering basic counselling for pregnant women. Furthermore, health workers’ perceptions of effort–reward-imbalance and professional quality of life will be assessed again at the end of the study to evaluate the impact of the ASSET intervention not just on patient health outcomes but also on those tasked with implementing the intervention themselves. These results will inform recommendations on how mental health task shifting interventions may be more sustainably implemented in the future.

More so, our study takes forward proposals to widen the ‘equity lens’ applied to health system strengthening efforts [72]. That exposure to stressful and unhealthy working conditions is a key driver of health inequities has been a central insight from work on the social determinants of health [73, 74]. Explicitly considering the wellbeing of health workers alongside the outcomes for patients when designing and evaluating health policies and programs is an important first step in thinking equity ‘all the way through’.

To the best of our knowledge, our study is one of the first studies employing the ERI scale to measure perceived levels of work-related psychosocial stress in sub-Saharan Africa. Whereas a number of other tools have been used to examine levels of work satisfaction and/or burnout amongst South African health workers, the ERI scale offers an explanatory framework with a primary focus that is less on the emotional exhaustion experienced by workers but more on the perceived mismatch between extrinsic costs and gains in the workplace (modified by individual coping characteristics). Furthermore, whereas the resulting psychosocial stress may lead to burnout, it may also affect other stress-related health outcomes, not least as those exposed to a stressful psychosocial work environment may be less able to lead balanced and healthy lives [69, 75]. In the context of the enduring health workforce constraints in low-and middle-income countries, and the concurring national and international efforts to rely on lay health workers to mitigate some of these constraints, the ERI construct may offer an additional useful tool to monitor the impact of policies and programmes on working conditions. Whereas our study used dual English/Afrikaans and English/IsiXhosa questionnaires, future studies should validate Afrikaans and IsiXhosa versions of the ERI scale.

Nonetheless, our study also has a number of limitations. As one of a range of studies conducted in the context of the larger ASSET research project, recruitment for this study was restricted to groups of health workers associated with PHC facilities that were part of the ASSET pre-implementation phase or those linked to intervention sites. Not least since there remain great variations in the ways CHW programs are implemented across South Africa [27], this may mean that results are not generalisable to other healthcare facilities beyond Cape Town. A small sample size also meant that we may have been underpowered to show associations between key variables. Lastly, as the ERI and PROQOL questionnaires rely on self-reported data, there is also the risk of reporting biases due to social desirability or affectivity [33], although this was mitigated by the fact that questionnaires were anonymous and self-administered [76].

Whereas the qualitative interviews provided further depth and context to findings from the quantitative survey, they arguably come with their own limitations. Since our interviewees all worked in a small number of public health facilities located in low resource settings in the Western Cape, their work experiences will be shaped by their—and their patients—exposure to particularly high levels of poverty, unemployment, ill-health and crime. Moreover, interviews were conducted in the midst of significant changes to the roles of lay health workers, which has, at least temporarily, introduced an additional set of uncertainties and strain, especially for lay health workers themselves.

It should be noted that, although we used semi-structured interview guides, the depth of discussions and range of topics covered varied across participants. Some participants will have likely been hesitant to raise what might be perceived as complaints about their workplace, or to appear overly critical of our planned intervention, especially when talking to academic researchers in positions of relative privilege. In addition, our own expectations and framing of the study will have inevitably further contributed to the setting of boundaries in terms of the issues that interviewees felt comfortable to raise, as well as the themes that we present in our findings. As a result, our data provides only a partial—and context-specific—picture of health workers’ experiences of their work environments.

Lastly, it is important to note that the research was conducted before the start of the COVID-19 pandemic. Cape Town quickly emerged as one of the focal points of the pandemic on the continent, and both primary healthcare facilities and community health workers played a key role in screening, testing and prevention efforts [77]. Increased workloads, risk of occupational exposure, and the mental and physical strain caused by successive infection waves are likely to only further exacerbate frontline health workers’ experiences of work-related stress, and call for future studies of the long-term impact of the pandemic burden.

## Conclusion

Our study findings demonstrate a high prevalence of work-related psychosocial stress psychosocial stress among professional and lay health workers providing care to perinatal women in the Western Cape. This was accompanied by moderate levels of Burnout and Compassion Fatigue. That results did not significantly differ between lay and professional health workers should be seen as an additional warning sign: although our initial hypothesis was informed by concerns over the impact of the significant changes to CHW programmes currently underway in South Africa, our study highlights that the perception of a mismatch between efforts spent and rewards gained is also widespread among professional health workers. Our results have already informed the design and evaluation of the task shifting intervention implemented as part of the wider ASSET research project. Furthermore, they add to the evidence base that local and international policymakers should take into account when designing strategies to improve population health and health equity: ensuring adequate working conditions and fostering the wellbeing of health workers are crucial preconditions to ensuring the sustainability of programmes and the quality of care they can deliver.

## Supplementary Information

**Additional file 1**. Demographic, Effort-Reward Imbalance (ERI) and Professional Quality of Life (PROQOL) questionnaires.

## Data Availability

The datasets generated and/or analysed during the current study are not publicly available due to the risk to the individual privacy of the study participants but are available from the corresponding author on reasonable request.

## Abbreviations

ANC: Antenatal care
BANC: Basic antenatal care
BO: Burnout
CF: Compassion fatigue
CBS: Community-based services
CS: Compassion satisfaction
CHW: Community health worker
CMD: Common mental disorders
DoH: Department of health
ERI: Effort–reward imbalance
HIV: Human immunodeficiency virus
HSS: Health systems strengthening
MOU: Midwife obstetric unit
NPO: Non-profit organisation
OTL: Outreach team leader
PHC: Primary healthcare
PROQOL: Professional quality of life
TB: Tuberculosis
WHO: World Health Organization
