# Supplementary material for: Exploring effort–reward imbalance and professional quality of life among health workers in Cape Town, South Africa: a mixed-methods study
Source: Glob Health Res Policy. 2022 Mar 1;7:7. doi: 10.1186/s41256-022-00242-6 (PMC8885139; doi:10.1186/s41256-022-00242-6)
Supplement: Supplementary file 1 — Additional file 1. Demographic, Effort-Reward Imbalance (ERI) and Professional Quality of Life (PROQOL) questionnaires. [file 41256_2022_242_MOESM1_ESM.docx]

**Demographic, Effort-Reward Imbalance (ERI) and Professional Quality of Life (PROQOL) questionnaires**

| Facility | |  | | Date |  |
| --- | --- | --- | --- | --- | --- |
| 1 | Age/ *Oudedom* |  | DOB/ *Geboorte datum* | | DD-MM-YYYY |
| 2 | Gender/ *Geslag* | | - Female/ *Vrou* - Male/ *Manlik* | | |
| 3 | Home language/ Huistaal | | - English - Afrikaans - IsiXhosa - Other (specify) ………………………………… | | |
| 4 | Population Group/ *Bevolkingsgroep* | | - Coloured - Black South African - Black other …………………………………. - Other (specify) ………………………………… | | |
| 5 | Religion/ Godsdiens | | - Christian/ *Christen* - Muslim/ *Moslem* - None - Other(specify) …………………………………. | | |
| 6 | Level of education | | - Grade 11-12 - Certificate - Diploma - Other (specify) ………………………………… | | |
| 7 | Who do you work for? | | - Department of Health - NGO (specify) ………………….……………… - Other (specify) ………………………………… | | |
| 8 | What job do you perform?  *Watter werk doen jy?* | |  | | |
| 9 | How long have you been in your current position?/  *Hoe lank is jy nou in jou huidige pos?* | | - Less than 6 months - 6-12 months - 1-2 years - 2-5 years - 5-10 years - More than 10 years | | |
| 10 | Do you have any mental health training?/  *Het jy enige opleiding in geestesgesondheid?* | | - Yes - No | | |
| 11 | What type of mental health training have you have?  *Watter soort opleiding vir geestesgesondheid het jy gehad?* | | - No training/ *Geen opleiding* - Workshop/ *Werkswinkel* - Module in certificate/ *Module in sertifikaat* - Other (specify) ………………………………… | | |
| 12 | How many people live in your home?  *Hoeveel mense woon by jou huis?* | |  | | |
| 13 | How many people receive an income in your home?  *Hoeveel mense verdien ‘n inkomste by die huis?* | |  | | |
| 14 | What are the sources of income in your home? *(may select multiple sources)*  *Wat is die bronne van inkomste by die huis? (kan verskeie opsies kies)* | | - Wages or Salary/ *Lone of salaris* - Own business/ *Eie besigheid* - Spouse or partner/ *Eggenoot of vennoot* - Social grant/ *Maatskaplike toelae* - Family/ *Gesin* - Other (specify) ………………………………… | | |
| 15 | Does your household have the same income every month?  *Is die huishouding inkomste dieselfde elke maannd?* | | - Yes, every month/ *Ja, elke maand* - Yes, most months/ *Ja, meeste maande* - No, it varies a lot/ *Nee, dit verskil* - Not sure/don’t know | | |
| 16 | How many children do you have?  *Hoeveel kinders het jy?* | |  | | |
| 17 | Do you have a partner?  *Het jy ‘n lewensmaat?* | | - Yes - No partner (skip to Q22) | | |
| 18 | What type of relationship do you have? *Watter tipe verhouding het julle?* | | - Married - Stable partner - Casual partner | | |
| 19 | Do you and your partner live together?  *Woon julle saam?* | | - Yes - No - Some of the time/ *Soms* | | |
| 20 | Does your partner provide financial support?  *Ondersteun jou lewensmaat jou finansiel?* | | - Yes - No - Some of the time/ Soms | | |
| 21 | What is your partner’s level of education?  *Wat is jou lewens maat se opvoedingkundige vlak?* | | - Grade 11-12 - Certificate - Diploma - Other (specify) ……………………………… | | |
| 22 | How do you get to work?  *Hoe kom jy by die werk?* | | - Public transport - Lift - Own car - Walk - Other (specify) ………………………………… | | |
| 23 | Do you own any of the following types of property? *(may select multiple sources)*  *Besit jy enige sort eiendom? (kan verskeie opsies kies)* | | - No ownership/ Geen eienskape nie - Formal house/ *Formele huis* - Flat/council house/ *Woonstel* - Shack/informal dwelling/ *Informele woning* - Backyard dwelling/  *Agterplaas woning* - Other (specify) ………………….. | | |
| 24 | Do you pay rent?  *Betaal jy huur?* | | - Yes - No | | |

| **The next few questions are about the demands of your current job. For each of the questions below, respond by ticking 1 block only** | | **Strongly agree** | **Agree** | **Disagree** | **Strongly disagree** |
| --- | --- | --- | --- | --- | --- |
| 1 | I have constant time pressure due to a heavy workload. /*Ek het aanhoudende tyd druk as gevolg van swaar werklas/werklading.* |  |  |  |  |
| 2 | I have many disturbances and interruptions while performing my job./  *Ek het baie versteurings en onderbrekings terwyl ek my werk doen.* |  |  |  |  |
| 3 | Over the past few years, my job has become more and more demanding. /  *Oor die laaste paar jaar het my werk meer en meer veeleisend geword.* |  |  |  |  |
| 4 | I receive the respect I deserve from my superior or a respective relevant person./  *Ek ontvang die respek van my hoër geplaaste (“superior”) of ‘n toepaslike persoon.* |  |  |  |  |
| 5 | My job promotion prospects are poor. /*My vooruitsigte vir werksbevordering is swak.* |  |  |  |  |
| 6 | I have experienced or expect to experience an undesirable change in my work situation. /  *Ek het ‘n ongewenste verandering in my werk situasie ervaar of verwag om ‘n ongewenste verandering in my werk situasie te ondervind.* |  |  |  |  |
| 7 | My job security is poor. / *My werksekuriteit is swak.* |  |  |  |  |
| 8 | Considering all my efforts and achievements, I receive the respect and prestige I deserve at work./ *Met al my pogings en prestasies in ag geneem, ontvang ek die respek en prestige wat ek verdien by die werk.* |  |  |  |  |
| 9 | Considering all my efforts and achievements, my job promotion prospects are adequate./ *Met al my pogings en prestasies in ag geneem, is my werksbevordering (promotion) vooruitsigte voldoende.* |  |  |  |  |
| 10 | Considering all my efforts and achievements, my salary/income is adequate. /*Met al my pogings en prestasies in ag geneem, is my salaris/inkomste voldoende.* |  |  |  |  |
| 11 | I get easily overwhelmed by time pressures at work. /*Ek word maklik oorweldig deur tyd druk by die werk.* |  |  |  |  |
| 12 | As soon as I get up in the morning I start thinking about work problems. */Sodra ek in die oggend opstaan, dink ek onmiddelik aan werkprobleme.* |  |  |  |  |
| 13 | When I get home, I can easily relax and ‘switch off’ work. /*Wanneer ek by die huis kom, kan ek maklik ontspan en afskakel van werk.* |  |  |  |  |
| 14 | People close to me say I sacrifice too much for my work. /*Mense na aan my sê ek offer te veel op vir my werk.* |  |  |  |  |
| 15 | Work rarely lets me go, it is still on my mind when I go to bed. /Werk is nog steeds in my gedagtes wanneer ek bed toe gaan. |  |  |  |  |
| 16 | If I postpone something I was supposed to do today I’ll have trouble sleeping at night. */As ek iets uitstel wat ek vandag moes doen, sal ek sukkel om in die nag te slaap.* |  |  |  |  |

| **The next few questions are about your current work situation. Tick the block that honestly reflects how frequently you experienced these things in the last 30 days. /Oorweeg elke vraag oor jouself en jou huidige werksomstandighede. Merk die blokkie wat eerlik weerspieël hoe gereeld jy hierdie dinge ondervind het in die laaste 30 dae.** | | **Never/**  **Nooit** | **Rarely/**  **Selde** | **Sometime/**  **Somtyds** | **Often/**  **Dikwels** | **Very often/**  **Baie gereeld** |
| --- | --- | --- | --- | --- | --- | --- |
| 1 | I am happy /*Ek is gelukkig* |  |  |  |  |  |
| 2 | I am preoccupied with more than 1 person I help /*Ek is besig met meer as 1 persoon wat ek help* |  |  |  |  |  |
| 3 | I get satisfaction from being able to help people /*Ek is tevrede om mense te kan help* |  |  |  |  |  |
| 4 | I feel connected to others /*Ek voel geheg aan ander* |  |  |  |  |  |
| 5 | I jump or am startled by unexpected sounds /*Ek spring of skrik vir onverwagte geluide* |  |  |  |  |  |
| 6 | I feel invigorated after working with those I help /*Ek voel verfris nadat ek met diegene wat ek help, gewerk het.* |  |  |  |  |  |
| 7 | I find it difficult to separate my personal life from my work life /*Ek vind dit moeilik om my persoonlike lewe van my werkslewe te skei* |  |  |  |  |  |
| 8 | I am not as productive at work because I am losing sleep over traumatic experiences of a person I helped/ *Ek is nie so produktief by die werk nie want ek verloor slaap weens traumatiese ervarings van iemand wat ek gehelp het* |  |  |  |  |  |
| 9 | I think that I might have been affected by the traumatic stress of those I helped /*Ek dink dat ek miskien beïnvloed is deur die traumatise stres van diegene wat ek gehelp het* |  |  |  |  |  |
| 10 | I feel trapped by my job/ *Ek voel vasgevang deur my werk.* |  |  |  |  |  |
| 11 | Because of my work, I have felt "on edge" about various things /*As gevolg van my werk, het ek “on edge” (op die rand) gevoel oor verskillende dinge* |  |  |  |  |  |
| 12 | I like my work /*Ek hou van my werk* |  |  |  |  |  |
| 13 | I feel depressed because of the traumatic experiences of the people I help /*Ek voel depressief as gevolg van die traumatiese ervarings van die mense wat ek help* |  |  |  |  |  |
| 14 | I feel as though I am experiencing the trauma of someone I have helped /*Ek voel asof ek die trauma ervaar van iemand wat ek gehelp het* |  |  |  |  |  |
| 15 | I have beliefs that sustain me **/***Ek het oortuigings (beliefs) wat my ondersteun* |  |  |  |  |  |
| 16 | I am pleased with how I am able to keep up with techniques and protocols (a set of rules and guidelines) related to my work /*Ek is tevrede met hoe ek kan by hou met tegnieke en protokolle (‘n stel reëls en riglyne) in verband met my werk* |  |  |  |  |  |
| 17 | I am the person I always wanted to be /*Ek is die persoon wat ek nog altyd wou wees* |  |  |  |  |  |
| 18 | My work makes me feel satisfied /*My werk laat my tevrede voel* |  |  |  |  |  |
| 19 | I feel worn out because of my work /*Ek voel uitgeput as gevolg van my werk* |  |  |  |  |  |
| 20 | I have happy thoughts and feelings about those I help and how I could help them /*Ek het gelukkige gedagtes en gevoelens oor diegene wat ek help en hoe ek hulle kan help* |  |  |  |  |  |
| 21 | I feel overwhelmed because my workload seems endless */Ek voel oorweldig omdat my werklading lyk asof dit geen einde het nie* |  |  |  |  |  |
| 22 | I believe I can make a difference through my work /*Ek glo ek kan ‘n verskil maak deur my werk* |  |  |  |  |  |
| 23 | I avoid certain activities or situations because they remind me of frightening experiences of the people I help /*Ek vermy sekere aktiwiteite want dit herinner my aan angswekkende ervarings van die mense wat ek help* |  |  |  |  |  |
| 24 | I am proud of what I can do to help /*Ek is trots op wat ek kan doen om te help* |  |  |  |  |  |
| 25 | As a result of my work, I have intrusive, frightening thoughts /*As gevolg van my werk, het ek indringende en skrikwekkende gedagtes* |  |  |  |  |  |
| 26 | I feel "bogged down" by the system /*Ek voel ‘vasgevang’ deur die stelsel* |  |  |  |  |  |
| 27 | I have thoughts that I am a "success" in my job /*Ek dink dat ek ‘n ‘sukses’ in my werk is* |  |  |  |  |  |
| 28 | I can't recall important parts of my work with trauma victims /*Ek kan nie belangrike dele van my werk met trauma slagoffers onthou nie* |  |  |  |  |  |
| 29 | I am a very caring person /*Ek is ‘n mens wat baie omgee* |  |  |  |  |  |
| 30 | I am happy that I chose to do this work /*Ek is bly dat ek gekies het om hierdie werk te doen* |  |  |  |  |  |
